# Comparison of Outcomes Between All-Inside Single-Bundle and Double-Bundle Anterior Cruciate Ligament Reconstruction: A Retrospective Study

**DOI:** 10.5704/MOJ.2303.003

**Published:** 2023-03

**Authors:** R Novriansyah, W Sundoko, A Wibowo, FS Putra

**Affiliations:** 1 Department of Orthopaedic Surgery, Universitas Diponegoro, Semarang, Indonesia; 2 Department of Orthopaedic Surgery, Universitas Indonesia, Jakarta, Indonesia

**Keywords:** ACL rupture, all-inside single-bundle ACL reconstruction, clinical outcomes, double-bundle ACL reconstruction, patient-reported outcomes

## Abstract

**Introduction:**

ACL rupture is the most common type of knee injury. The All-inside ACL reconstruction procedure features some distinguished components including closed-socket tunnels with less bone expulsion, double suspensory fixation, and smaller incisions. We aimed to compare the outcomes between the All-inside Single-bundle and the Double-bundle ACL reconstruction techniques.

**Materials and methods:**

This study was a retrospective study which analysed the patient-reported and the clinical outcomes on patients who underwent ACL reconstruction between January and December 2020 at Dr Kariadi General Hospital Semarang, Indonesia. We compared the patient-reported and the clinical outcomes at 6- and 12-month follow-ups between the All-inside Single-bundle and the Double-bundle groups. The patient-reported outcomes were determined using the IKDC and Tegner-Lysholm scores while the clinical outcomes included the measurement of Thigh Circumference, Single Hop test, Anterior Drawer test, Lachman test, Range of motion, and the patient’s level of return to sport.

**Results:**

A total of 24 subjects were divided into two groups, namely the All-inside Single-bundle and the Double-bundle groups, consisting of 12 subjects in each group. Most of the subjects were male in both groups, including 9 (75%) subjects in the All-inside Single-bundle group, and 11 (91.67%) subjects in the Double-bundle group. The mean age of the subjects were 25.75±7.57 years old in the All-inside Single-bundle group, and 24.5±6.87 years old in the Double-bundle group. In terms of the side of the knee that suffered the most injuries in both groups were the right knees. The result of the patient-reported outcomes using IKDC and Tegner-Lysholm scores showed no statistically significant differences in both groups at 6- and 12-month follow-ups (p=0.864; p=0.293 and p=0.589; p=0.233, respectively). The results of clinical assessments at 6- and 12-month follow-ups also showed no statistically significant differences in both groups.

**Conclusion:**

Our study showed no significant differences in the patient-reported and the clinical outcomes between the All-inside Single-bundle and the Double-bundle ACL reconstruction techniques at 6- and 12-month follow-ups.

## Introduction

Anterior cruciate ligament (ACL) rupture is the most common type of knee injury, with an estimated incidence of 30 to 78 per 100,000 person-years^1,2^. Most ACL ruptures are caused during the exercises which involves pivoting, rapid starting, braking, impacting or colliding^3^. These injuries are commonly related to contact sports (basketball, soccer, etc)^4^. A previous study in the USA found that around 250,000 individuals suffered from ACL rupture annually^5^.

ACL reconstruction remains the standard operative treatment for ACL rupture^1^. Single-bundle (SB) and Double-bundle (DB) reconstruction are currently the most applied techniques, both of which aim to restore the function and anatomy of the knee joint^6,7^. While the SB reconstruction technique restores the anterior stability of the knee, it cannot restore the rotational function of the knee joint^6^. Previous studies believed that the DB reconstruction technique is superior to the SB technique as it reconstructs both the Anteromedial (AM) and the Posterolateral (PL) bundles of the ACL^7^. The DB technique improves pivot shift resistance and increases rotational knee control contrasted with the SB technique^1,6,7^.

The All-inside ACL reconstruction technique features some distinguished components including closed-socket tunnels with less bone expulsion, double suspensory fixation, and smaller incisions^8,9^. Several studies showed both controversies and potential benefits of the All-inside technique compared to the standard reconstruction technique^8^. Previous studies revealed that patients who underwent All-inside ACL reconstruction had lower post-op VAS pain scores compared to those who underwent the standard ACL reconstruction^10,11^. However, other studies which assessed other variables including IKDC, KSS, Lysholm, and Tegner showed no significant difference between the All-inside ACL and the standard ACL groups^11^. To our knowledge, there have not been previous studies which specifically compare the outcomes between the All-inside Single-bundle and the Double-bundle ACL reconstruction techniques. The present study aimed to compare the outcomes between the All-inside Single-bundle and the Double-bundle ACL reconstruction techniques.

## Materials and Methods

This study was an analytical observational study using retrospective cohort design comparing the outcomes between the All-inside Single-bundle and the Double-bundle ACL reconstruction techniques which took place at Dr Kariadi General Hospital Semarang, Indonesia. The size of the sample in this study was calculated using the Cochran’s formula, as followed^12^:

n=Zα2pqd2

n = minimum size sample required

Z = Z score, based on the desired α value

α = confidence level

d = fault tolerance

p = proportion of cases studied in the population

q = 1-p, i.e., the proportion for the occurrence of an event

The Z value was adjusted for the α value. The confidence level used in this study was 5%, hence the α value was 0.05 and the Zα value of 1.96 was obtained. This study used fault tolerance of 20%, which meant that the accuracy rate was 80%. Therefore, the d value was 0.2. Since p was unknown, the largest p was used (p=0.5). Since this study used the largest p, then the q value was 1-p (1-0.5=0.5). Based on these calculations, the result was 24.01 which was rounded to 24. Therefore, 24 patients were needed as subjects of this study.

We collected the sample using the consecutive sampling technique, which was a sampling technique in which every subject meeting the criteria of inclusion was selected until the required sample size was achieved.

The subjects of the study were all patients who underwent ACL reconstruction between January and December 2020 at Dr Kariadi General Hospital Semarang, Indonesia. The reconstruction technique selected, either All-inside Single-bundle or Double-bundle, was determined based on each patient’s level of sport activity, lifestyle, and occupation. All patients underwent similar rehabilitation protocol according to the D’Amato Protocol for ACL Injury which was standardised in our center^13^. The inclusion criteria were: (1) Participant aged ≥18 years; (2) Participant who was diagnosed with isolated ACL injury; and (3) Participant who underwent ACL reconstruction (All-inside Single-bundle or Double-bundle techniques) at our centre. Meanwhile, the exclusion criteria were: (1) Participant who refused to participate in the study; (2) Participant who underwent previous ACL reconstruction in the involved knee; and (3) Participant who had concomitant injury, including meniscal injury, posterior cruciate ligament injury, collateral ligament injuries, fracture or dislocation, and osteoarthritis.

Characteristics of the subjects such as gender, age, occupation, sport activity, side of the impacted knee were obtained from each patient’s medical record. The primary outcome of this study was the patient-reported outcomes assessed by using the International Knee Documentation Committee (IKDC) and Tegner-Lysholm scores. The IKDC and Tegner-Lysholm scores were acquired from the patient’s medical record since those were routinely assessed by the physician in our centre. The IKDC questionnaire consisted of 11 items relating to the knee symptoms, function, and sports activities. The scores ranged from 0 to 100 points. In the other hand, the Tegner-Lysholm questionnaire consisted of items relating to limp, the need for support, pain, instability, locking, swelling, stair climbing, and squatting. The scores ranged from 0 to 100 points.

The secondary outcome of this study was the results of various clinical investigations including thigh circumference, Single Hop test, Anterior Drawer test, Lachman test, Range of motion, and the patient’s level of return to sport. Thigh circumference was measured using a measuring tape at the half-distance between the greater trochanter and the lateral condyle of the femur. It included the thigh circumference of the healthy and impacted knee as well as their deficits. Meanwhile, the Single Hop, Lachman, and anterior drawer tests were graded as positive or negative. The range of motion of the knee was measured using a goniometer. Lastly, the patient’s level of return to sport was graded into four categories, namely 0: not able to return to sport, 1: mild activity (walking), 2: non-contact sport (jogging, passing), and 3: return to competitive sport^14,15^. We collected all of the data from the patients’ medical records at pre-operative assessment, 6th and 12th monthly follow-up.

All of the ACL reconstruction procedures were conducted by one consultant hip and knee orthopaedic surgeon, so there was no bias in data collection. For all of the subjects, we used one type of graft, which was hamstring graft, to avoid bias. In the All-inside technique, we harvested the graft with a tendon stripper. The required graft length was 6cm, and the diameter was 7cm. Different from standard anatomical ACL reconstruction techniques, the All-inside technique uses the Retro Construction System to create tunnels with a retrograde drill. An autograft tunnel was drilled on the femur based on the identified ACL footprint sites through the accessory medial portal. Meanwhile, in a double bundle ACL reconstruction, we tried to reproduce the anteromedial (AM) and posterolateral (PL) bundle position and function of the native ligament. The required graft lengths were 6-7cm each, and the diameters were 7-8cm each.

We compared the data between the pre-operative assessment and the follow-ups. The pre-operative IKDC and Tegner-Lysholm scores were compared to the scores at 6- and 12-month follow-ups. We also compared the IKDC and Tegner-Lysholm scores between the All-inside Single-bundle and the Double-bundle groups at 6- and 12-month follow-ups. Meanwhile, the secondary outcome including the clinical assessments was also compared between the All-inside Single-bundle and the Double-bundle groups at 6- and 12-month follow-ups.

Ethical approval was obtained from the Health Research Ethics Committee of Dr Kariadi General Hospital Semarang (No. 342/EC/KEPK-RSDK/2021).

We performed a descriptive analysis on the characteristics of the subjects. The data of the characteristics of the subjects were presented as total number (n) or mean and standard deviation (SD), as appropriate. IKDC and Tegner-Lysholm scores were presented as mean and standard deviation (SD). For the clinical assessment, thigh circumference and range of motion were presented as mean and standard deviation (SD). Meanwhile, the Anterior Drawer test, Lachman test, Single Hop test, and return to sport level were presented as total number (n). For statistical analysis, we performed normality test using Shapiro-Wilk test. Subsequently, we used the paired T-test for the data with normal distribution to compare the pre-operative and follow-up data. Meanwhile, to compare the data of 6- and 12-month follow-up between the two groups, we used the Independent T-test for the data with normal distribution. In the other hand, we used Mann-Whitney U-test, and Fisher χ-square test for the data with abnormal distribution. Differences with a P-value of <0.05 were regarded as statistically significant. Data analysis was performed using SPSS® version 28.0 [IBM, New York, United States].

## Results

A total of 24 subjects who met the inclusion and exclusion criteria were recruited into this study. The subjects were then divided into two groups, namely the All-inside Single-bundle and the Double-bundle groups, with each group consisting of 12 subjects. The characteristics of the subjects of the study are listed in (Table I). The majority of the subjects were male patients in both groups, including 9 (75%) male subjects in the All-inside Single-bundle group, and 11 (91.67%) male subjects in the Double-bundle group. The mean age of the subjects was 25.75±7.57 years old in the All-inside Single-bundle group, and 24.5±6.87 years old in the Double-bundle group. Furthermore, the majority of the subjects were college students, consisting of 4 (33.33%) subjects in the All-inside Single-bundle group, and 5 (41.67%) subjects in the Double-bundle group. In terms of sport activity, the most common type of sport was running (33.33%) in the All-inside Single-bundle group, and football (58.33%) in the Double-bundle group. Meanwhile, in terms of the side of the knee that suffered the most injuries in both groups were the right knees, which were 9 (75%) subjects in the All-inside Single-bundle group, and 8 (66.67%) in the Double-bundle group.

**Table I:** Characteristics of the subjects

	All-inside Single-bundle	Double-bundle
Number of patients, n (%)	12	12
Gender, n (%)		
Male	9 (75)	11 (91,67)
Female	3 (25)	1 (8,33)
Mean age, yr (mean±SD)	25.75±7.57	24.5±6.87
Occupation, n (%)		
College student	4 (33.33)	5 (41.67)
Private employee	4 (33.33)	3 (25)
Civil servant	2 (16.67)	0 (0)
Self-employed	2 (16.67)	1 (8.33)
Healthcare provider	0 (0)	2 (16.67)
Student	0 (0)	1 (8.33)
Sport activity, n (%)		
Football	3 (25)	7 (58.33)
Running	4 (33.33)	0 (0)
Basketball	1 (8.33)	2 (16.67)
Volleyball	0 (0)	1 (8.33)
Handball	0 (0)	1 (8.33)
Swimming	1 (8.33)	0 (0)
Wushu	1 (8.33)	0 (0)
Pencak Silat	0 (0)	1 (8.33)
Cycling	1 (8.33)	0 (0)
Futsal	1 (8.33)	0 (0)
Impacted knee, n (%)		
Right knee	9 (75)	8 (66,67)
Left knee	3 (25)	4 (33,33)

The primary outcome of this study was the patient-reported inputs using the IKDC and Tegner-Lysholm scores. The result of the patient-reported outcomes at pre-operative assessment and follow-ups can be seen in (Table II and III). The IKDC and Tegner-Lysholm scores at 6-month follow-up were significantly improved in both groups compared to the pre-operative assessment (p<0.001). The IKDC scores increased from 49.9±12.64 to 65.9±18.92, while the Tegner-Lysholm scores increased from 44.50±15.11 to 71.17±22.89 in the All-inside Single-bundle group at 6-month follow-up. In the Double-bundle group, the IKDC scores increased from 42.52±10.78 to 67.24±19.02, while the Tegner-Lysholm scores increased from 48.42±22.08 to 80.25±18.16 at 6-month follow-up. At 12-month follow-up, the IKDC and Tegner-Lysholm scores were also significantly improved in both groups compared to the pre-operative assessment (p<0.001). The IKDC scores increased from 49.9±12.64 to 82.17±9.24, while the Tegner-Lysholm scores increased from 44.50±15.11 to 85±13.48 in the All-inside Single-bundle group at 12-month follow-up. In the Double-bundle group, the IKDC scores increased from 42.52±10.78 to 84.08±7.84, while the Tegner-Lysholm scores increased from 48.42±22.08 to 90.33±6.70 at 12-month follow-up.

**Table II:** Comparison of IKDC and Tegner-Lysholm Scores before the operation and at six months follow-up in the All-inside Single-bundle and Double-bundle groups

Groups	IKDC Score (mean±SD)		P-value	Tegner-Lysholm TScore (mean±SD)		P-value
	Pre-operative	Six months follow-up		Pre-operative	Six months follow-up	
All-inside Single-bundle	49.9±12.64	65.9±18.92	<0.001	44.50±15.11	71.17±22.89	<0.001
Double-bundle	42.52±10.78	67.24±19.02	<0.001	48.42±22.08	80.25±18.16	<0.001

**Table III:** Comparison of IKDC and Tegner-Lysholm Scores before the operation and at 12-month follow-up in the All-inside Single-bundle and Double-bundle groups

Groups	IKDC Score (mean±SD)		P-value	Tegner-Lysholm Score (mean±SD)		P-value
	Pre-operative	12-month follow-up		Pre-operative	12-month follow-up	
All-inside Single-bundle	49.9±12.64	82.17±9.24	<0.001	44.50±15.11	85±13.48	<0.001
Double-bundle	42.52±10.78	84.08±7.84	<0.001	48.42±22.08	90.33±6.70	<0.001

**Fig. 1:**
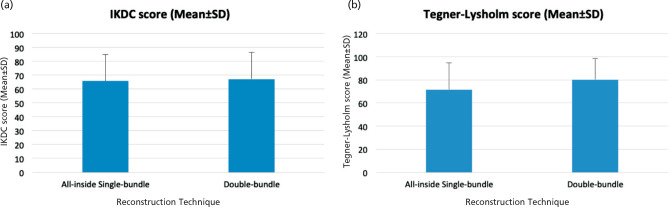
(a) IKDC Scores between the All-inside Single-bundle and Double-bundle groups at six months follow-up. (b) Tegner-Lysholm Scores between the All-inside Single-bundle and Double-bundle groups at six months follow-up.

**Fig. 2:**
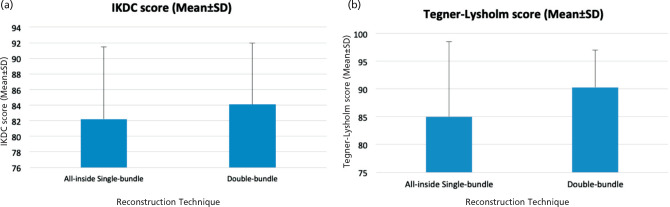
(a) IKDC Scores between the All-inside Single-bundle and Double-bundle groups at 12-months follow-up. (b) Tegner-Lysholm Scores between the All-inside Single-bundle and Double-bundle groups at 12-months follow-up.

The result of the patient-reported outcomes using the IKDC and Tegner-Lysholm scores at 6- and 12-month follow-ups can be seen in (Table IV and V). The Independent T-test showed that there were no statistically significant differences in the IKDC and Tegner-Lysholm scores between the All-inside Single-bundle and the Double-bundle group at 6-month follow-up (p=0.864; p=0.293, respectively). At 12-month follow-up, the result also showed no statistically significant differences in the IKDC and Tegner-Lysholm scores between the two groups (p=0.589; p=0.233, respectively). The result of the Independent T-test on the patient-reported outcomes using the IKDC and Tegner-Lysholm scores at 6- and 12-month follow-ups can be seen in (Fig. 1 and 2).

**Table IV:** Comparison of IKDC and Tegner-Lysholm Scores between the All-inside Single-bundle and Double-bundle groups at six months follow-up

	All-inside Single-bundle	Double-bundle	P-value
IKDC score (mean±SD)	65.9±18.92	67.24±19.02	0.864
Tegner-Lysholm score (mean±SD)	71.17±22.89	80.25±18.16	0.293

**Table V:** Comparison of IKDC and Tegner-Lysholm Scores between the All-inside Single-bundle and Double-bundle groups at 12-month follow-up

	All-inside Single-bundle	Double-bundle	P-value
IKDC score (mean±SD)	82.17 ± 9.24	84.08 ± 7.84	0.589
Tegner-Lysholm score (mean±SD)	85 ± 13.48	90.33 ± 6.70	0.233

The secondary outcome of this study was the clinical outcomes including Thigh Circumference, Single Hop test, Anterior Drawer test, Lachman test, Range of motion, and the patient’s level of return to sport. Table IV and V summarises the results of clinical assessments in both groups at 6- and 12-month follow-ups. The Independent T-test showed that there were no statistically significant differences in thigh circumference at 6- and 12-month follow-ups (p=0.628 and p=409, respectively). Regarding the range of motion, the Mann-Whitney U test showed no statistically significant differences at 6- and 12-month follow-ups (p=0.385 and p=1.000, respectively). Furthermore, the Fisher χ-square test also showed no statistically significant differences in Anterior Drawer, Lachman, and Single Hop tests at 6- and 12-month follow-ups (p=1.000; p=1.000; p=1.000, and p=0.478; p=0,478; p=0,478, respectively). In terms of return to sport level, the Mann-Whitney U test showed that there were no statistically significant differences at 6- and 12-month follow-ups (p=0.782 and p=0,623, respectively).

**Table VI:** Comparison of clinical assessments between the All-inside Single-bundle and Double-bundle groups at six months follow-up

	All-inside Single-bundle	Double-bundle	P-value
Thigh circumference, cm			0.628
Healthy knee	43.67±5.26	42.67±2.90	
Impacted knee	42.75±5.68	41.92±3.12	
Deficits	0.92±0.90	0.75±0.75	
Range of motion, degree	120±19.19	125.83±13.62	0.385
Anterior Drawer test, n (%)			1.000
Positive	4 (33.33)	3 (25)	
Negative	8 (66.67)	9 (75)	
Lachman test, n (%)			1.000
Positive	4 (33.33)	3 (25)	
Negative	8 (66.67)	9 (75)	
Single Hop test, n (%)			1.000
Positive	8 (66.67)	8 (66.67)	
Negative	4 (33.33)	4 (33.33)	
Return to sport level, n (%)			0.782
0: not able to return to sport	0 (0)	0 (0)	
1: mild activity (walking)	5 (41.67)	4 (33.33)	
2: non-contact sport (jogging, passing)	4 (33.33)	5 (41.67)	
3: return to competitive sport	3 (25)	3 (25)	

**Table VII:** Comparison of Clinical Assessments between the All-inside Single-bundle and Double-bundle groups at 12-month follow-up

	All-inside Single-bundle	Double-bundle	P-value
Thigh circumference, cm			0.409
Healthy knee	43.67±5.26	42.67±2.90	
Impacted knee	43.25±5.50	42.42±2.91	
Deficits	0.42±0.52	0.25±0.45	
Range of motion, degree	135±0.0	135±0.0	1.000
Anterior Drawer test, n (%)			0.478
Positive	2 (16.67)	0 (0)	
Negative	10 (83.33)	12 (100)	
Lachman test, n (%)			0.478
Positive	2 (16.67)	0 (0)	
Negative	10 (83.33)	12 (100)	
Single Hop test, n (%)			0.478
Positive	0 (0)	2 (16.67)	
Negative	12 (100)	10 (83.33)	
Return to sport level, n (%)			0.623
0: not able to return to sport	0 (0)	0 (0)	
1: mild activity (walking)	0 (0)	0 (0)	
2: non-contact sport (jogging, passing)	3 (25)	2 (16.67)	
3: return to competitive sport	9 (75)	10 (83.33)	

## Discussion

The result of our study showed no statistically significant differences in the patient-reported and the clinical outcomes between the All-inside Single-bundle and the Double-bundle groups at 6- and 12-month follow ups. There have not been previous studies comparing the outcomes between the All-inside Single-bundle and the Double-bundle ACL techniques. Torkaman *et al* (2016) evaluated the results of traditional SB and DB techniques using Lysholm score, and found no significant differences between the two groups^16^. In the other hand, Chen *et al* (2015) conducted meta-analysis of randomised controlled trials comparing traditional SB and DB techniques, and found no significant difference in the IKDC scores^1^. Chen *et al* (2020) compared the clinical impacts of individualised anatomic SB and DB, and showed no significant difference between the two groups in terms of the IKDC, Lysholm and Tegner scores^6^. Another study by Zhang *et al* (2014) also showed no statistically significant differences between SB and DB ACL reconstructions on Tegner, and Lysholm scores at 24-month follow-up^17^.

In this study, we also performed physical examinations related to the function of ACL including Lachman, Anterior Drawer, and Single Hop tests. Our results showed no significant differences in all of those tests between the two groups. Oh *et al* (2020) showed that anterior tibial translation measured with the anterior drawer test was superior in the DB technique^18^. Their results showed that the DB technique was more effective than the SB technique in controlling anterior stability^18^. A single hamstring tendon harvest provides adequate length to serve as the autograft when tripled or quadrupled^19^. Previous study utilising a human cadaveric model showed that utilising a single hamstring tendon could re-establish anterior tibial translation to within 1.3mm of the native ACL in response to a 134-N load^18^. Previous studies revealed that the utilisation of a single hamstring graft in the all-inside technique resulted in similar knee stability compared to the native ACL and similar clinical outcomes compared to standard techniques^8,9^. Chen *et al* (2020) showed no significant difference in the Lachman test according to the clinical examination at the final follow-up between the SB dan DB ACL reconstructions^6^.

In terms of return to sport level, our results showed no significant difference between the All-inside single-bundle and Double-bundle techniques. Most of the subjects (33.33 to 41.67%) in our study were able to return to non-contact sport (jogging, passing, etc.) during the 6-month follow-up. Some of the subjects (25%) were able to return to competitive level. However, during the 12-month follow-up, more subjects achieved to return to competitive levels (75% in the All-inside single-bundle group, and 83.33% in the Double-bundle group). Ardern *et al* conducted a meta-analysis of studies evaluating return-to-sport level after ACL reconstructions, and found that 63% of the subjects were able to return to their preinjury level of sport, and 44% of them were able to return to competitive sport^20^. The period for return to sport after ACL reconstruction is variable depending on the patient’s tolerance for the exercise, the surgeon preferences, and the physical needs of the sport^21,22^. Hensler *et al* (2012) recommended that return to sport started at 9 to 12 months after surgery due to the higher graft loads after ACL reconstruction^21^. In the other hand, Shelbourne and Nitz (1990) suggested that return to sport initiated at 3 to 6 months after surgery^23^. However, they stated that the decision of return to sports was dependent based on achieving near normal strength in the impacted limb, full knee extension, no knee effusion, and completion of the rehabilitation program^23^.

In this study, there was no significant difference in postoperative side-to-side difference in thigh circumference between the two groups. It was shown that in All-inside Single-bundle group the difference was slightly more than in Double-bundle group, but this did not significantly affect the clinical outcomes. For post-operative range of motion, there was also no significant difference between the All-inside Single-bundle and the Double-bundle groups.

Our results showed significantly improved patient-reported outcomes in both groups at 6- and 12-month follow-ups compared to the pre-operative assessment, which indicated that both techniques comparably improved the knee joint function. However, our results showed no statistically significant differences in the patient-reported and the clinical outcomes between the two groups, which indicated that there was no superiority between the two techniques. Either the All-inside Single-bundle or the Double-bundle techniques can be equally considered based on the patient’s indications such as their level of sport activity, lifestyle, and occupation^24^.

The All-inside ACL reconstruction technique is attracting a lot of interest due to its special features of using a single tendon autograft as compared to two tendon autografts used in the conventional technique^25^. The spared hamstring tendon should leave the leg with a greater hamstring strength, leading to an improved functional outcome^23^. However, this technique poses several challenges^8^. It is imperative to highlight the surgical complexity of this technique, therefore this is considered as a surgeon-dependent technique^8,26^. One of the common challenges faced by surgeons when performing the all-inside ACL reconstruction technique is drilling the femoral socket using the AM portal technique^8^. Some commonly reported difficulties associated with AM portal drilling include the hyperflexed knee position required to properly drill the femoral socket^26^. Another technical challenge includes a suture passing wires management in a little space under arthroscopy view^26^. Another matter which needs to be taken into account when deciding to perform this technique is the cost^8^. One study conducted in France compared the cost difference between an all-inside and standard ACL techniques, and found that the cost of the all-inside ACL technique was 18% more expensive than the standard technique^8,27^. This increase in cost was mostly caused by the single use equipment required for the retrograde drilling and suture pass in the all-inside ACL technique^8,27^. However, in our country, the equipment used are all reusable, therefore multiple usage could damage the equipment eventually. This matter could be a limitation in performing this surgical technique in our country.

On the other hand, the Double-bundle ACL reconstruction technique are considered to be able to restore the original anatomical structure of the impacted ACL more closely than the conventional Single-bundle reconstruction technique^6^. Previous studies also showed that the DB technique has superior results related to the knee stability, which was more effective in controlling anterior stability compared to the conventional SB technique^18^. However, this technique is not without disadvantages, such as longer operation time, increased risk of bone bridge fractures, and challenge in revision surgery^28^. The DB technique is often used in athletes in demanding level of sports activity (cutting, jumping, or pivoting sports), or patients with heavy work occupation, and high-risk lifestyle^24^.

Overall, our results showed that both the All-inside Single-bundle and the Double-bundle reconstruction techniques provided similar good outcomes. Therefore, in considering the option of the reconstruction technique, it is the utmost importance for physician to determine a surgical technique based on each patient’s characteristics and demands.

Our study was the first study to compare outcomes between the All-inside Single-bundle and the Double-bundle ACL reconstruction techniques. Therefore, our results might give new insights to surgeon and patients in the application of surgical technique selection depending on individual patient’s demands and physical conditions. However, our study has several limitations. Firstly, the relatively small sample size. However, we calculated the sample size using Cochran’s formula with the accuracy rate of 80% which was adequate for this study. Secondly, we did not randomise the subjects and blinding the researcher during the study due to our limited resource. Lastly, our follow-up duration was relatively short. Nevertheless, our study was the first study aimed to compare the outcomes between the All-inside Single-bundle and the Double-bundle ACL reconstruction techniques.

A future study with a larger sample size is needed to substantiate our current findings. Further studies using different methods are also needed, such as prospective study and double-blind randomisation study, to further analyse the outcomes. Finally, longer follow-up duration might also be needed to significantly support our findings.

## Conclusion

Our study showed no significant differences in the patient-reported and the clinical outcomes between the All-inside Single-bundle and the Double-bundle ACL reconstruction techniques at 6- and 12-month follow-ups.
